# Feasibility and acceptability of a self-management intervention supporting return to work for women with breast cancer

**DOI:** 10.1177/03080226251319900

**Published:** 2025-03-20

**Authors:** Naomi Algeo, Kathleen Bennett, Louise Brennan, Deirdre Connolly

**Affiliations:** 1 Discipline of Occupational Therapy, Trinity College Dublin, Dublin, Ireland; 2 Trinity St. James’s Cancer Institute, St. James’s Hospital, Dublin, Ireland; 3 Data Science Centre, School of Population Health, RCSI University of Medicine and Health Sciences, Dublin, Ireland; 4 Discipline of Physiotherapy, Trinity College Dublin, Dublin, Ireland

**Keywords:** cancer survivorship, vocational rehabilitation, employment, breast cancer, occupational therapy, intervention development

## Abstract

**Introduction:**

Increased cancer survivorship has prompted focus on optimising quality of life, including work reintegration. Despite frequently cited return-to-work barriers for women with breast cancer, there are no conclusive work-focused interventions for this cohort. The aim of this study was to assess feasibility and acceptability of a self-management intervention supporting return-to-work for women with breast cancer.

**Methods:**

A mixed-methods single-arm feasibility study evaluated intervention feasibility. Participants completed an online occupational therapy-led ‘Work and Cancer’ intervention over 6 weeks. Feasibility was determined by recruitment, adherence, retention and acceptability. Acceptability was also assessed through semi-structured interviews. Data were analysed using descriptive statistics and thematic analysis.

**Results:**

Ten out of twelve participants who were approached, participated in the study. Retention and adherence was 100% and 90%, respectively. Every participant completed pre- and post-measures. Qualitative data indicated online and group format as enablers to intervention completion and juggling employment as a barrier.

**Conclusion:**

It is feasible to recruit and retain participants to the ‘Work and Cancer’ intervention which was widely accepted by women with breast cancer. Large-scale evaluation will determine intervention effectiveness on work and health-related outcomes.

## Introduction

Breast cancer is common, and accounted for an estimated 2.26 million new cases worldwide in 2020 (GLOBOCAN, 2020). Increased survivorship has prompted focus on optimising quality of life (QoL) for those living with and beyond cancer, including reintegration into employment. Work participation offers several benefits: increased QoL, a sense of ‘normalcy’, increased self-esteem and reduced social isolation (Schmidt et al., 2019; van Maarschalkerweerd et al., 2020). Despite this, return-to-work rates are generally lower in breast cancer, where the 1-year time-point is commonly viewed as a milestone, although this rate varies (Balak et al., 2008; Chaker et al., 2015). There are several factors which could explain this. For example, those with breast cancer are more likely to receive chemotherapy, compared to those with prostate cancer (National Cancer Registry Ireland, 2024). Chemotherapy is known to be a return-to-work barrier due to side effects such as cancer-related fatigue, cognitive dysfunction and psychological distress all of which can persist and impact work ability (Islam et al., 2014). In addition, women living with and beyond breast cancer may experience breast and arm comorbidities post-surgery, which are strongly associated with being on sick leave (Wennman-Larsen et al., 2013).

Legislative systems can also influence return-to-work. For example, in the Republic of Ireland, there is a right to reasonable work accommodations; however, paid sick leave has only recently become mandated. A recent qualitative-descriptive study in the Republic of Ireland, highlighted limited awareness amongst women with breast cancer of employment legislation in the context of a cancer diagnosis, and that experiences of sick leave and pay entitlements varied (Algeo et al., 2021a). In addition, while most women did not report any discrimination on returning to the workplace, there were examples of indirect discrimination where unfair expectations were being placed on women by colleagues.

Despite frequently cited return-to-work barriers for women with breast cancer (Sun et al., 2016), a systematic review identified that there are no conclusive work-focused interventions for this cohort (Algeo et al., 2021b). This is despite women expressing a desire for additional resources to support work outcomes. For example, an interpretative phenomenological study highlighted the desire of women living with and beyond breast cancer for healthcare professional support on functional and work ability (MacLennan et al., 2022). Indeed, women living with and beyond breast cancer, up to 10 years post-diagnosis, expressed regret that they had not received oral and written information regarding the return-to-work process for themselves and their employers throughout their cancer trajectory (van Maarschalkerweerd et al., 2020).

Internationally, there are limited return-to-work supports available; however, the effectiveness of such interventions has not been formally established. For example, in the United Kingdom, Macmillan offer training under the Macmillan-at-Work intervention which includes expert training, consultancy, information and support and resources including e-newsletter and free work and cancer toolkit, yet there is no evidence available to demonstrate efficacy (Macmillan, 2019). There are several written resources on returning to work after a cancer diagnosis available in Canada (Beck and Amin, 2019), Australia (Cancer Council, 2019), and the United States (American Cancer Society, 2021), however, these are not tailored to the individual and not necessarily applicable to someone living with and beyond a cancer diagnosis in Ireland, where legislative systems are different.

While there remains a lack of effective and methodologically rigorous rehabilitation intervention studies to support work outcomes for those living with and beyond cancer specifically (Algeo et al., 2021b; de Boer et al., 2015), occupational therapists have, for decades, been addressing employment needs, where interventions have been proven to positively influence employment outcomes for other cohorts with chronic conditions (Désiron et al., 2011). Occupational therapists have key skills, including self-management, that can support those with physical and/or mental health conditions to overcome barriers to engage in meaningful occupation, and commence, resume, or retain employment (Royal College of Occupational Therapists, 2021).

In response to recommendations for further research in the area, and in line with research priorities to address the role of occupational therapy in supporting chronic conditions, participation in everyday life (Mackenzie et al., 2018), and self-management (Royal College of Occupational Therapists, 2021), we developed an occupational therapy-led self-management intervention guided by the Medical Research Council (MRC) framework for developing and evaluating complex interventions (Skivington et al., 2021). Typically, a developed intervention can be further refined through exploratory methods such as a feasibility study (Craig et al., 2008). Under the MRC framework, a feasibility study prior to a definitive trial is recommended as it can identify potential problems which could undermine acceptability and delivery of an intervention such as issues with recruitment, adherence, retention or acceptability of the intervention (O’Cathain et al., 2015). The aim of this study was to assess feasibility and acceptability of the *Work and Cancer* intervention to support return-to-work for women living with and beyond breast cancer. The research questions are

1. What length of time is required to complete participant recruitment and what are the reasons for declining participation?
2. What is the adherence to and completion (retention) rate of the intervention?
3. Are outcome measures acceptable to women living with and beyond breast cancer, and what is the completion rate?
4. Is the intervention acceptable to women living with and beyond breast cancer?

## Methods

*Study design*: A parallel mixed-methods design consisting of a single-arm feasibility study and qualitative descriptive design was conducted between February and April 2021. Parallel mixed designs involve two or more parallel quantitative and qualitative components conducted either simultaneously or with minimal time lapse (Creswell and Plano Clark, 2011). A single-arm design was chosen as exploration of the feasibility of randomisation, or use of a control was not an outcome of this research. A qualitative-descriptive design was integrated into the study through the facilitation of individual semi-structured interviews, approximately one-week post-intervention.

*Participants*: Potentially eligible participants were identified by a gatekeeper in a single community cancer support centre in the Republic of Ireland and contacted via telephone. Purposive sampling was used in participant selection and involves choosing a sample based on similar or identical traits, as per inclusion criteria: (i) women who were living with and beyond breast cancer, (ii) were in employment at time of diagnosis, and (iii) had either returned to work and self-reported to be struggling at work or were aiming to return to work within the following 6 months from recruitment. All participants were required to have an internet-enabled device that could support the Zoom Video Communications Inc. platform. Similar studies exploring online psycho-social interventions have recruited up to eight participants per group to manage group dynamics in an online format (Lemma and Fonagy, 2013; Muscara et al., 2020). Therefore, it was aimed to recruit up to 10 participants to account for potential attrition which has been commonly estimated at 20% for digital interventions (Foster et al., 2016; Howarth et al., 2018). As this was a feasibility study in design, a sample size calculation was not conducted. Sample sizes in feasibility studies are typically small and vary depending on the objectives of the study, although typically start from 10 per group (Lewis et al., 2021). While data saturation for the qualitative-descriptive design could also be considered where it has been recommended (Bowen, 2008), to interview a sample of ten cases followed by at least a further three cases to determine if any new themes emerge, the overall aim of this study was to establish the feasibility of a work-focused intervention, and therefore sample size criteria for a feasibility study were prioritised, and data saturation did not apply in this case.

*Participant recruitment*: Data were collected between February and April 2021. In order to avoid a relationship between the researcher and potential participant during recruitment, a gatekeeper from a single community cancer support centre approached potentially eligible participants from their database who was invited to contact the research team directly. A participant information leaflet (PIL), consent form and outcome measures were issued prior to the intervention which took place over a 6-week period. Participants were made aware in the PIL that the intervention was part of a research study and were informed of the research objectives. Those interested in taking part were then invited to contact the researcher to express interest in participation if they wished to proceed with the study. Participants were invited to pose questions to the researcher on any aspect of the study prior to signing consent. Sessions 1–5 were group-based and facilitated weekly, and session 6 was one-to-one, and organised at a mutually convenient time for 1 hour. Semi-structured interviews were conducted one-week post-intervention and via Zoom Communications Inc. platform.

*Intervention*: The *Work and Cancer* intervention is described in accordance with TIDieR checklist criteria (Supplemental Material 3). It is a 6-week online intervention underpinned by self-management theory, delivered using Zoom Video Communications Inc. platform. Five sessions are group-based, with a final 1:1 session with a registered occupational therapist who is an author of this paper (Table 1). Each group session commences with weekly topic content via PowerPoint and closes with goal setting. A 5-minute comfort break was offered between education content and goal setting. Emphasis is made to understand side effects and developing strategies to manage these at work. Each session was interactive, where participants could pose questions or make comments/reflections at any stage of the educational component.

**Table 1.** *Work and cancer* intervention.

Week	Title	Session goal(s)	Content	Facilitator (s)	Format	Length	Setting
Week 1	Introduction to the Programme	▪ To familiarise group with programme, SMART goal setting and one another	▪ Outline of Programme ▪ Introductions ▪ SMART goal setting	Occupational Therapist	Group	90 minutes	Online via Zoom
Week 2	Employment Rights and Entitlements after Cancer	▪ To understand rights entitlements and grants related to work in the context of cancer. ▪ To understand reasonable accommodations and how they might apply to particular job roles.	▪ Employment Rights & Entitlements ▪ SMART goal setting	Occupational Therapist and Community Welfare Officer			
Week 3	Managing Cancer-Related Fatigue and Cognitive Changes in the Workplace	▪ To understand causes and patterns of cancer-related fatigue and brainfog. ▪ To understand principles in energy conservation and how to manage brainfog in the context of work.	▪ Understanding Cancer-related Fatigue & Application to Workplace ▪ Understanding Cognitive Changes & Application to Workplace ▪ SMART goal setting	Occupational Therapist			
Week 4	Communicating Effectively with your Employer, Colleagues and Family	▪ To learn strategies in which to navigate unwanted conversations, medical disclosure, navigating a return-to-work plan with your employer.	▪ Re-engaging with your Workplace ▪ Negotiating a Return-to-Work Plan/Maintenance Plan ▪ SMART goal setting	Occupational Therapist			
Week 5	Managing Your Mental Health and Physical Side-Effects in the Workplace	▪ To develop strategies in managing stress and anxiety in work. ▪ To understand ergonomics ▪ To understand physical side-effects and how they could be managed in work.	▪ Managing Stress & Anxiety in the Workplace ▪ Managing Physical Side-Effects in the Workplace & Ergonomics ▪ SMART goal setting	Occupational Therapist and Physiotherapist			
Week 6	Developing a Return-to-Work Roadmap	▪ Occupational therapist to complete occupational analysis. ▪ Occupational therapist to develop Return-to work/work maintenance plan and letter of reasonable accommodations	▪ Occupational Analysis ▪ Return to Work Plan & Letter of Reasonable Accommodations ▪ Assessing Readiness to Return to Work ▪ SMART goal setting	Occupational Therapist	Individual	~60 minutes	

Self-efficacy is a factor that can influence an individual’s self-management. Based on social cognitive theory (Bandura, 1986), self-efficacy is the belief or confidence that individuals have that they can perform a required behaviour to produce a desired outcome (Bandura, 1997). Greater levels of self-efficacy can lead to greater levels of perceived control over one’s actions and behaviours. The four key components which can influence self-efficacy were embedded into the *Work and Cancer* intervention to support self-management (Lorig and Holman, 2003) as follows:

▪ *Modelling*: Throughout the intervention, participants discussed how they applied information learned to change behaviours.
▪ *Performance mastery:* As part of action planning, SMART goal setting was embedded at the end of each session of the intervention, where participants set occupation-based goals in relation to their return to work.
▪ *Verbal/Social persuasion:* Support and positive feedback from peers as well as the presence of others can encourage changes in behaviour (Mukhtar et al., 2012). Discussion among the group was encouraged throughout the group-based sessions.
▪ *Physiological feedback/Interpreting symptoms:* Each session of the intervention included education on specific topics (e.g. cancer-related fatigue, cognitive dysfunction). Education was combined with strategies to address self-management of these symptoms. Participants could then consider trialling and applying these strategies in their own lives and in the workplace.

Prior to the intervention, participants were mailed a 155-page ‘*Work and Cancer*’ handbook providing supplementary information on each session topic, additional exercises and presentation slides for each session. They were encouraged to read each session’s corresponding chapter, prior to attending each week, and could use the resource to look back on if needed in the future. The handbook was developed based on previous phases of this research, and co-designed by women with breast cancer, healthcare professionals and other key stakeholders (Algeo et al., 2022). As the intervention remains under testing, the handbook is not widely available at this time.

*Data collection and instruments of data collection*: Feasibility involves exploring features such as recruitment (how many potential participants who are approached, participated), adherence (attendance at the intervention sessions) and retention to (completion of) an intervention as well as intervention acceptability (Orsmond and Cohn, 2015). The feasibility of measuring outcomes was also examined. Four data collection tools were issued via post 1-week pre-intervention:

▪ Locally developed questionnaire assessing employment and participant demographics (e.g. job role, baseline working hours, length of sick leave)
▪ European Organisation for Research and Treatment of Cancer Quality of Life Questionnaire (EORTC-QLQ-C30) Version 3.0 (Aaronson et al., 1993): The 30-item EORTC-QLQ-C30 is an internationally recognised tool which is both reliable and valid for assessing cancer-specific QoL in those living with and beyond cancer (Aaronson et al., 1993).
▪ Breast Cancer Subscale (QLQBR23) (Sprangers et al., 1996) measured breast cancer-specific QoL. The 23-item QLQ-BR23 is a breast cancer-specific module of the EORTC QLQ-C30 and has been widely tested for its reliability and validity cross culturally (Sprangers et al., 1996).
▪ Worker Role Functioning Questionnaire 2.0 (WRFQ) (Abma et al., 2013) was issued to those who had returned to work. The 27-item WRFQ is a widely used tool for the assessment of health-related work functioning (Abma et al., 2018), and has been found to be a reliable and valid instrument in working individuals living with and beyond cancer (Dorland et al., 2021).

All measures were repeated 1-week post-intervention and completion rates recorded. Semi-structured interviews, guided by piloted interview schedules (Supplemental Material 4), were also conducted one-week post-intervention. Interviews were audio-recorded and conducted by the first author (Occupational Therapist, PhD Candidate) who recorded field-notes throughout. No one other than the first author and participant was present during interviews. Participants were reminded of the objectives of the research. Interviews averaged 56 minutes. The first author independently completed transcription and data were managed using NVivo12©. Several strategies were used to enhance credibility of findings including member-checking, encouraging honesty, scrutiny of the research and the researcher background.

### Data analysis

*Quantitative data*: Descriptive statistics regarding participant recruitment, adherence and retention of study and intervention procedures were tracked throughout the intervention and recorded on a Microsoft^®^ Excel spreadsheet and presented in the form of frequencies, percentages and means, where appropriate. In the absence of formal guidance, progression criteria in this study were determined based on a feasibility study of a similar online cancer survivorship intervention (O’Neill et al., 2021). Feasibility to proceed to a definitive evaluation of the *Work and Cancer* intervention was determined by the acceptability by women living with and beyond breast cancer as well as the following progression criteria: ⩾50% of eligible participants recruited, mean of ⩾80% adherence to the online intervention, and ⩾83% retention at post-intervention assessment. Progression criteria are often under-reported in feasibility studies often leading to variations in the interpretation of findings (Mbuagbaw et al., 2019). Sessions which were partially attended were considered adhered to if >50% of the session was attended. Demographic characteristics were summarised using descriptive statistics (e.g. frequencies and percentages for categorical variables, and means and SDs for continuous variables) with IBM SPSS Statistics for Mac, Version 23 (United Kingdom).

*Qualitative data*: Interviews were audio-recorded and transcribed verbatim. Transcripts were uploaded onto NVivo12© and qualitative data were analysed using thematic analysis (Braun and Clarke, 2022) which comprised of six steps: First, (i) transcripts were read and re-read to enhance familiarity and search for emerging patterns, and then (ii) data of interest was highlighted, and initial codes developed. A second coder, a co-author experienced in qualitative research, reviewed a sample of initial coding. Open coding was used where no pre-set codes had been determined. Where any deviations occurred in coding between the two authors, discussion was prompted, with a third researcher available for any unresolved disagreements, although this was not required. Codes were (iii) grouped into themes and these themes were then (iv) reviewed and refined. Themes were (v) defined and labelled, before (vi) a final report was drafted.

### Pursuing quality in qualitative research

Reporting of the feasibility study follows the extended COSORT checklist for pilot and feasibility studies (Supplemental Material 1). The qualitative-descriptive component follows the COREQ criteria (Supplemental Material 2). Several techniques were used throughout the conduct of the qualitative-descriptive design to enhance the trustworthiness of the research. For example, member-checking, providing a detailed description of study context to support transferability, use of an audit trail and peer scrutiny. In addition, the first author of this study was also the interventionalist and recognised potential researcher bias for acceptability of the intervention. Several measures were put in place to attempt to bracket any biases. These included, but were not limited to, (i) identifying assumptions and preconceptions at the outset of this research and throughout using memos, and (ii) ensuring an additional researcher checks transcripts, codes and final themes’.

## Results

### Demographics

The mean age of participants was 47.6 years (SD: 5.06). Mean working hours pre-diagnosis was 34.75 hours (SD: 8.9), and mean sick leave was 15.73 months (SD: 6.33). Full participant demographics captured pre-intervention are presented in Table 2.

**Table 2.** Participant demographics.

Identifier	Age	Occupational group^a^	Working hours pre-diagnosis	RTW (pre-intervention)	Sick leave^b^
P1	48	Managers and executives	50	No	>11 months
P2	54	Social work and related occupations	37	Yes	23 months
P3	50	Clerical and office workers	22	No	>14 months
P4	43	Business and commerce	35	Yes	22 months
P5	54	Teachers	27.5	Yes	11 months
P6	45	Business and commerce	45	No	>6 months
P7	37	Clerical and office workers	37	No	>25 months
P8	48	Health and related workers	26	No	>20 months
P9	49	Clerical and office workers	28	Yes	8 months
P10	48	Social work and related occupations	40	Yes	16 months

a Occupational group is structured as per Central Statistics Office (CSO) Occupation Groups definitions (CSO, 2002).

b Where a participant had not yet returned to work pre-intervention, sick leave was recorded with ‘>’ to recognise sick leave period at that time.

### Feasibility

**Figure 1.**
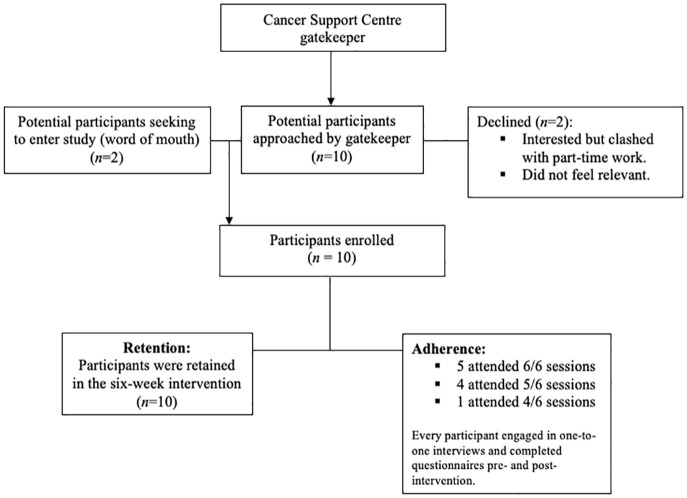
Participant recruitment, adherence and retention.

*Recruitment*: Ten out of twelve individuals meeting the inclusion criteria approached were recruited into the intervention over a 3-week period between January and February 2021 via a gatekeeper at a single cancer support centre (Figure 1; recruitment rate 83%). Two participants learned about the intervention via word of mouth and approached the researcher directly. Reasons for non-participation included clashing with part-time work and feeling that the intervention was not relevant to their situation.

*Adherence*: The mean attendance of the intervention was 90% (range 67–100%). Half of participants attended all six sessions. Where sessions were missed, reasons for non-participation included medical appointments (*n* = 3), clashed with work (*n* = 2), and bereavement (*n* = 1). Every participant engaged in an interview and completed each questionnaires pre- and post-intervention. There were five sessions of which participants partially attended due to conflicting events and were able to do so because of the online format.

*Retention*: Every participant completed the intervention, interviews and pre- and post-intervention questionnaires (100%, *n* = 10).

### Outcome measures

Completion of outcomes measures was considered good with only 1.48% and 0.19% missing items for the WRFQ and EORTC-QLQ-C30, respectively. There were no missing items for the QLQ-BR23.

*WRFQ*: Findings from the WRFQ are presented in Table 3. There were observed increases in median scores in work scheduling demands, physical demands and social demands.

**Table 3.** WRFQ pre- and post-intervention median values (IQR).

Outcome	Median (IQR)	
	Pre-intervention (*n* = 5)	Post-intervention (*n* = 5)
Work scheduling demands	75 (42.5, 97.5)	85 (52.5, 90.0)
Output demands	87.5 (53.5, 100.0)	80.4 (66.1, 94.6)
Physical demands	60.4 (27.1, 90.0)	87.5 (62.5, 90,8)
Mental demands	87.5 (31.3, 89.6)	83.3 (54.2, 86.3)
Social demands	87.5 (56.3, 100.0)	95.8 (72.9, 100.0)
Overall	90 (44.0, 94.9)	86.4 (61.3, 90.8)

Higher scores indicate higher work function.

*EORTC-QLQ-C30*: Category scores from the EORTC-QLQ-C30 are presented in Table 4. Every category score improved (Summary, Functional and Symptom scores), with the exception of Global Health Status where there was a decrease in post-intervention score. Examining EORTC-QLQ-C30 functional scales, there were increases in emotional and cognitive functioning scores, no changes in physical or social functioning, and a decrease in role functioning (Table 5).

**Table 4.** Comparison of changes in Median Values (IQR) of EORTC-QLQ-C30 category scores pre-and post-intervention.

Category	Pre-intervention (*n* = 10) Median (IQR)	Post-intervention (*n* = 10) Median (IQR)	Change in Median
Global Health Status Scores	62.5 (47.9, 77.1)	54.17 (50.0, 77.1)	8.33
Functional Scores	62.23 (55.6, 85.0)	70.09 (58.9, 75.0)	7.86
Symptoms Scores	26.93 (10.3, 37.2)	25.64 (16.7, 37.8)	1.29
Summary Scores	62.5 (57.1, 90.9)	63.3 (55.2, 87.5)	0.80

Higher scores for global health status and functional scores indicate higher level of functioning. However, higher scores of symptom scales indicate higher levels of issues. Higher summary score indicates higher QoL.

**Table 5.** EORTC-QLQ-C30 pre- and post-intervention median values (IQR).

Outcomes	Median (IQR)	
	Pre-intervention (*n* = 10)	Post-intervention (*n* = 10)
Functional scales		
Physical functioning	80 (66.7, 81.7)	80 (71.7, 86.7)
Role functioning	66.7 (25.0, 83.3)	50.0 (41.7, 91.7)
Social functioning	58.3 (29.2, 100.0)	58.3 (45.8, 87.5)
Emotional functioning	58.3 (45.8, 83.3)	70.8 (39.6, 83.3)
Cognitive functioning	50.0 (45.8, 70.8)	58.3 (29.2, 66.7)
Global health status		
Global health status	62.5 (47.9, 77.1)	54.2 (50.0, 77.1)
Symptom scales		
Fatigue	33.3 (22.3, 58.4)	44.3 (22.3, 69.4)
Nausea and vomiting	0.0 (0.0, 16.7)	8.3 (0.0, 16.7)
Pain	50.0 (25.0, 75.0)	41.7 (29.2, 50.0)
Single items		
Dyspnoea	0.0 (0.0, 33.3)	0.0 (0.0, 33.3)
Insomnia	50.0 (25.0, 100.0)	66.7 (33.3, 75.0)
Appetite loss	0.0 (0.0, 0.0)	0.0 (0.0, 0.0)
Constipation	0.0 (0.0, 75.0)	0.0 (0.0, 41.7)
Diarrhoea	0.0 (0.0, 0.0)	0.0 (0.0, 8.3)
Financial difficulties	33.3 (0.0, 66.7)	33.3 (0.0, 33.3)

Higher scores for functional scales and global health status indicate higher level of functioning. However, higher scores of symptom scales and single items indicate higher levels of issues.

*QLQ-BR23*: Examining QLQ-BR23 data, higher function was observed in every scale except future perspective and arm symptoms which did not change (Table 6).

**Table 6.** QLQ-BR23 pre- and post-intervention median values (IQR).

Outcome	Median (IQR)	
	Pre-intervention (*n* = 10)	Post-intervention (*n* = 10)
Functional scales		
Body image	37.5 (8.3, 77.1)	41.7 (6.2, 66.7)
Sexual functioning	8.3 (0.0, 20.8)	16.7 (0.0, 37.5)
Future perspective	83.3 (58.3, 83.3)	83.3 (66.7, 83.3)
Symptom scales		
Systemic therapy side effects	23.8 (14.3, 36.9)	19.0 (11.9, 23.4)
Breast symptoms	41.7 (16.7, 52.1)	29.2 (14.6, 37.5)
Arm symptoms	33.3 (19.5, 55.7)	33.3 (22.3, 55.7)

Higher scores for functional scales indicate higher level of functioning. However, higher scores of symptom scales indicate higher levels of issues.

### Acceptability

**Figure 2.**
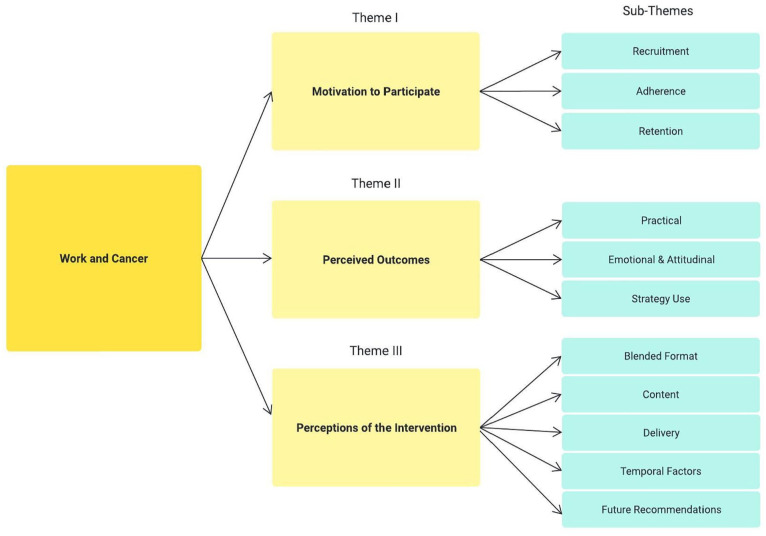
Key themes and sub-themes.

Three key themes emerged from data analysis and comprised several sub-themes (Figure 2).

### Theme I: Motivation to participate: Curiosity and comradery

Motivations for enrolment included interest and ‘*curiosity*’ (P5; P6). Another participant, who had yet to return to work, reflected on a gap in post-acute care, highlighting the intervention as an opportunity to ask questions in the area:

I thought this is a really worthwhile thing to look at . . . there [is] constant questioning once you’ve finished the regular hospital appointments, you’ve nobody really to ask. (P8)

Motivations to complete the intervention included interest in the topic and feeling part of a group where ‘*you*’*re not alone in all of this*’ (P5):

It was very interesting, so I suppose that was my motivation to stay on every week . . . you weren’t thinking ‘Oh God, is this ever going to end!?’ and most of the time you were thinking, ‘Gosh, is the time up already?’. It just seemed to fly by. (P9)

### Theme II: Perceived outcomes: ‘I can manage this’

Every participant discussed how they had changed their approach in transitioning back to or maintaining their job role with varied examples provided throughout. This included taking control of a phased RTW, enhancing awareness of ergonomics or creating a framework in navigating RTW. One participant discussed how their perspective of what a phased RTW looked like, changed post-intervention:

Only by talking to you guys and others in the group, that I realised, ‘Well listen, a phased return – it does not mean six weeks’, you know? It’s longer than that. So, the programme definitely has brought perspective, and actually made me acutely aware that, you know, I need to think of myself. (P6)

*Practical*: Practical outcomes refer to when information/theory learned through the intervention is applied in a real-life context. Most participants (*n* = 8) requested a letter of personalised reasonable accommodations for their employer. Reasons for non-request included autonomy in role and feeling that it had been too long post-RTW. Words used to describe the letter included ‘*powerful*’ (P2), ‘*it just gives you that peace of mind*’ (P6), and ‘*supportive*’ (P8). All participants received a RTW Plan or Work Maintenance Plan. Words used to describe the reports included ‘*worthwhile*’ (P2), ‘*clear*’ (P7), ‘*invaluable*’ (P9), and ‘*really helpful*’ (P8).

Participants also discussed how increased knowledge of ergonomics led to environmental changes in their workplace. One participant who had returned to work found benefit from learning about ergonomics and reflected on how she could adapt her workplace:

I have begun to look carefully around me. Even the layout of how my stuff is organized and where I store things on shelves and on my desk. You know, even the logistics of getting my books and equipment and resources to and from school. (P5)

Several participants also discussed new knowledge on financial entitlements and grants that they were not aware of prior to the intervention:

The fact that I was told about the invalidity [benefit] has lifted a huge weight off of my shoulders because I was forcing myself to be ready by a certain time, by a certain date. Whereas now, I don’t have that pressure. So, now when I actually go back to work, it’s because I will be physically and emotionally ready which is amazing. (P7)

*Emotional and attitudinal*: Participants reflected on several perceived emotional and attitudinal changes including a new sense of empowerment, reduced feelings of anxiety around RTW and validation of their RTW experiences. Several participants reflected on feelings of empowerment in self-managing their condition:

I have to say the biggest takeaway for me has been the feeling of ‘I can manage this’ . . . I got, what I called a nugget or a gem from every session that I thought ‘I can do something with this’ . . . So, the practicality of it was instrumental in changing my perception of my return to work which was negative. (P8)

One participant discussed how the intervention and peer feedback increased awareness of their condition and caused an attitudinal shift in how they viewed their RTW:

Only by talking to you guys, and others in the group, that I realised, ‘Well listen, a phased return – it does not mean six weeks’, you know? It can be longer. So, the programme has definitely broadened my perspective, and actually made me acutely aware that, you know, I need to think of myself. (P6)

For women who had yet to RTW, the most reported perceived outcome from the intervention was reduced anxiety navigating the RTW process often enabled through increased confidence and knowledge:

I don’t feel as anxious . . . it’s like, ‘I’m going to go back to work, and I have no expectations of myself for the first two, three weeks until I find my feet’. And I suppose it wouldn’t have dawned on me before that that was ok. (P8)

*Strategy use*: Finally, participants discussed how the intervention influenced self-management of their treatment- and disease-related side effects, using strategies. In particular, participants discussed using strategies and reflective exercises to self-manage their fatigue and cognitive changes:

I’m still actually doing that energy diary . . . when I’m working I use a notepad and in the back I’ll kind of write how I’m feeling that morning and how I’m feeling by the end of the work day just giving me an idea of trying to gauge how I am on those days. (P4)

Following the intervention, participants also reflected on the use of strategies in managing their cognitive function. For example, on planning their work tasks ahead to ensure they were not too cognitively heavy:

If there was talk of assigning me work, I was very mindful to say, ‘Listen, start me off with something that’s well defined and not as cognitively taxing so I can see how I how I can accommodate it’. (P6)

### Theme III: Perceptions of the intervention: ‘A One-Stop Shop’

Every participant used positive terminology to describe their overall impressions of the intervention. Words such as ‘supportive’ (P3), ‘very comprehensive’ (P5), ‘really special’ (P8), ‘empowering’ (P7) and ‘hugely beneficial’ (P2) were used to describe the intervention, with every participant reporting content to be relevant in the context of work, with applicability to all job roles. One participant reflected on the intervention being unique in that it pulls information on work and cancer together:

There’s a lot written about [work and cancer] but there’s nothing that pulls it all together in a one-stop shop package like this. (P8)

*Blended format*: Every participant reported preference for the blended format, suggesting a range of advantages for having both group-based and one-to-one sessions. Participants reflected on ‘*the benefit of learning from each other*’ (P1), the acknowledgement that ‘*you*’*re not alone*’ (P2) and ‘*interaction*’ (P9) as key benefits to the group-based sessions. Emphasis was placed on the personalised component of the one-to-one session which was described by participants as ‘*personalised*’ (P1), and the opportunity for ‘*individual support*’ (P4).

*Delivery*: Every participant reported the online delivery of the intervention acceptable, describing it as ‘*very easy*’ (P1), ‘*so much more flexible*’ (P7) and ‘*second nature*’ (P6). Reasons for acceptability included the ability to use online features (e.g. raise hand function and mute) to minimise interruption being able to complete the intervention in the comfort of one’s own home and eliminating commuting. The use of Zoom as a platform to deliver the intervention was warmly received and was described as ‘*very easy to use*’ (P9), ‘*mostly fool-proof*’ (P7), ‘*hassle-free*’ (P10) and ‘*Much more straightforward*’ (P2). If the intervention were to be hosted face-to-face in the future, every participant indicated preference for community-based settings. Specifically, cancer support centres were suggested by eight of the participants who associated words such as ‘*relaxed*’ (P6) ‘*feels more like home*’ (P7) and ‘*comforting*’ (P10) with the setting:

I do think a more relaxed setting kind of thing where you can make your cup of tea and coffee and, you know, you have maybe more beautiful surroundings. (P5)

The acute setting, on the other hand, was associated with unwanted memories of treatment which could be ‘*triggering for some people*’ (P8) and a setting associated ‘*with going into scans and check-ups and anxiety*’ (P4).

*Temporal Factors*: Most participants reported that the intervention length of six weeks was ‘just right’:

I think [the intervention length is] just about right. I think any shorter and we may not have come on the journey, or it might have been a bit more magpie-ish so different participants homing in on different pieces but not getting the whole framework and not being brought through a process of thinking. (P1)

It wasn’t too long for sure. It flew by for us. For me anyway. No, I think it was probably just right because everything that was in it was relevant and I can’t think of any topic that you didn’t cover that could have been in there. (P2)

There were mixed feelings on session length (‘*Perfect*’ (P8), ‘*Just enough*’ (P6), ‘*Okay*’ (P5)), although no participant suggested decreasing length. Most participants stated an amendment to session length could be increased by 15–30 minutes online to allow for a short break in between, and provide further opportunities to socially interact,

It’s hard to sit still for too long and you don’t want to make it go on too long but I do think that [sessions] were short as in we were often rushed towards the end and that was really when people were getting going and that was really when people were sort of sharing stories, experiences and opinions. (P4)

I think if it was face-to-face, it would naturally run longer because people are going to be telling stories and I think that that’s one thing that you do less, that I’ve noticed that you do less online. (P8)

*Future of the intervention*: Every participant indicated a desire for the intervention to be rolled out nationally in the future. The potential for future roll-out of the *Work and Cancer* intervention was described as ‘*so necessary*’ (P1), ‘*very important*’ (P10) and ‘*so beneficial*’ (P8) in the context of what was described as ‘*an under-resourced area*’ (P8) where ‘*there really wasn*’*t anything*’ (P1).

Several minor amendments were suggested to embed into future research of the intervention, including outcome measures and the issuing of documents such as the consent forms, outcome measures. Most participants described the outcome measures as user-friendly with words used to describe them as ‘*easy*’ (P7), ‘*easy to understand*’ (P9) and ‘*there was nothing difficult about it*’ (P2). Despite this, there were some minor amendments suggested. For the locally developed questionnaire, participants discussed removing the word ‘fully’ from the question ‘Do you feel fully ready to return to work?’ as well as to provide more options in answering that question. For example, to include a ‘*between partially and yes*’ (P3).

## Discussion

The objective of this study was to evaluate a work-focused intervention for feasibility and acceptability among women living with and beyond breast cancer. The findings indicate that the intervention was widely accepted by participants and that it is feasible to recruit and retain participants in the 6-week online intervention. Therefore, for women living with and beyond breast cancer, a large-scale evaluation is warranted to determine intervention effectiveness on work and health-related outcomes.

Adherence to the intervention and retention were considered high at 90% and 100%, respectively, although this is not unusual for women living with and beyond breast cancer. A Cochrane review exploring home-based multidimensional survivorship interventions for women living with and beyond breast cancer, found adherence ranged from 58 to 100% (Cheng et al., 2017), with four of the eight studies recording adherence, reporting >90% attendance. There may be several reasons for this. Group-based interventions have previously been recommended in increasing adherence due to enhanced motivation and peer support (Michael et al., 2021) and may have been a factor of the intervention which encouraged adherence. This was echoed by qualitative findings, which indicated a preference for a group format and has been observed as a preference over one-to-one interventions in other studies for those living with and beyond cancer (Oswald et al., 2021; Rabin et al., 2013). The intervention also took place during a significant restriction period in public health in response to the COVID-19 pandemic, and this could have encouraged adherence, where there were fewer competing activities. Despite this, intervention feedback was consistently positive, with participants citing interest in the topic and feeling part of a group as motivators in completing the intervention. Women living with and beyond breast cancer typically seek out and utilise healthcare services more frequently than other cancer cohorts; however (Boland, 2018; Treanor et al., 2013), and therefore, there is the potential that feasibility may not be generalisable to other cancer cohorts. Future studies could explore the feasibility of the intervention with other cancer cohorts. In addition, research could explore the feasibility of a face-to-face format, particularly in a landscape post-pandemic. Promising feasibility findings however support the potential implementation of the *Work and Cancer* intervention online, pending outcomes of effectiveness in a definitive trial.

Beyond the intervention, completion of outcome measures pre- and post-intervention was also deemed to be feasible where participants reported measures to be easily understood and completed each relevant outcome measure, with 1.48% and 0.19% overall missing items for the WRFQ and EORTC-QLQ-C30/QLQ-BR23, respectively. This has been considered feasible in the past. While missing responses for items for the WRFQ are infrequently reported, some studies which do cite missing items have previously ranged from 2 to 3.5% (Ramada et al., 2014a, 2014b). Missing items using the EORTC-QLQ-C30 range from 0 to 8.6% (Apolone et al., 1998; Davda et al., 2021; Waldmann et al., 2013). In general, qualitative findings supported the use of instruments as acceptable. Therefore, use of both WRFQ, EORTC-QLQ-C30 and QLQ-BR23 could be considered feasible in future research in this context. Despite this, other outcome measures could be explored, where the appropriate selection of outcome measurements for an intervention can be challenging where close attention is paid to what outcome should be measured as well as how it should be measured (Coster, 2013).

Identifying suitable outcome measures for a RTW intervention for women living with and beyond breast cancer was also considered in this study. While the EORTC-QLQC30, QLQ-BR23 and WRFQ had high compliance rates and were perceived as acceptable by women living with and beyond breast cancer, qualitative findings suggested some potential gaps in outcome measurement that may be of importance. For example, qualitative findings highlighted perceived changes in self-efficacy, where women highlighted a new feeling of empowerment and attitudinal changes towards their RTW. This could be important in measuring as higher job self-efficacy is related to an earlier RTW, as well as a stronger predictor for a full RTW than work ability (Wolvers et al., 2018). The PROMIS^®^ Self-Efficacy for Managing Chronic Conditions is one outcome measure that has demonstrated internal consistency and cross-sectional validity (Gruber-Baldini et al., 2017), and could be applied to a cancer population. More specifically, work self-efficacy could be examined using an outcome measure such as the 19-item Return-to-Work Self-Efficacy Questionnaire (RTWSE) which has demonstrated reliability and adequate validity in a cancer population (Rosbjerg et al., 2021). This could be of particular use to participants who have yet to return to the workplace and were not in a position to complete the more detailed WRFQ for work outcomes.

Finally, overcoming barriers to the intervention should be considered in future implementation. Quantitative findings highlighted partial adherence to the intervention (67%) from one participant due to work commitments. The juggling of work roles was widely cited by participants in qualitative findings as a potential barrier in completing the intervention and is to be expected. Previous studies exploring supportive interventions for those living with and beyond cancer, cited work commitments as a barrier in attending a cancer survivorship intervention (Boland, 2018; Rabin et al., 2013). Despite this, the inclusion of those who have returned to work and self-report to be struggling is important. Evidence suggests that treatment and disease-related side effects can be chronic (Bodai and Tuso, 2015; Mokhatri-Hesari & Montazeri, 2020), and support is warranted for this cohort. As such, flexibility of intervention delivery outside of working hours might be considered, such as in the evening or at weekends if possible, as suggested by Lyons et al. (2015). No other barriers were discussed and instead emphasis placed on the online format as a facilitator to intervention completion.

*Implications for practice*: The *Work and Cancer* intervention is a 6-week self-management programme that aims to support work ability of women living with and beyond breast cancer. Occupational therapists are well placed to deliver the online intervention and, as suggested by participants in this study, eligibility of the intervention could be expanded to other cancer cohorts once evaluated. While MDT input was welcomed, strategic delivery of such input could be considered from a pragmatic implementation perspective. For example, it may be difficult to co-ordinate an occupational therapist in leading the intervention, alongside a community welfare officer and physiotherapist. Therefore, based on participant feedback, recorded video input could be considered from a physiotherapy perspective, where it may be otherwise difficult to offer personalised advice without formal assessment. Video education has been found to be feasible for a breast cancer cohort, although this was not physiotherapy-specific (Sulakvelidze et al., 2019). Despite this, there is emerging evidence exploring physiotherapy-specific video education for women living with and beyond cancer (Guloglu et al., 2022).

*Implications for future research*: Findings from this feasibility study indicate that the *Work and Cancer* intervention is feasible to implement, and acceptable to women living with and beyond breast cancer. Therefore, a large-scale randomised control trial evaluation is warranted to determine intervention effectiveness on work- and health-related outcomes. Elements of the intervention to be retained in future research studies include the work-focused content, 6-week duration and hybrid delivery of group-based sessions and one-to-one occupational therapy input. Factors that will be modified include the increase of session length and distribution of questionnaires, in the future development and testing of the *Work and Cancer* intervention. As the intervention was tested for feasibility using the online platform, there may be benefit in piloting the intervention face to face, in addition to the online format. It is becoming increasingly clear, as healthcare professionals and patients continue to navigate the pandemic, that variety in service delivery may be necessitated and needs to be evaluated.

The sample size was small in this feasibility study (*n* = 10); therefore, future piloting of the intervention is warranted to increase sample size to more accurately estimate a sample size for large-scale evaluation. Typically, an overall sample size of 30 is recommended as a ‘general rule of thumb’ for good practice (Lancaster et al., 2004: 308). Determining the primary outcome measure prior to sample size calculation is also required. In this study, a potential gap in outcomes to be measured was identified, where qualitative findings highlighted the importance of self-efficacy in ability to RTW or maintain work which was an outcome not explored previously.

*Strengths and limitations*: This study offers insight into the feasibility and acceptability of the ‘Work and Cancer’ intervention. The study design is strengthened with the qualitative-descriptive component of the study exploring uncertainties prior to a larger evaluation. Limitations also exist, however. Findings of this preliminary study should be cautiously interpreted, acknowledging the small sample size. In addition, data were collected by the interventionalist which may have influenced responses. One occupational therapist facilitated the intervention which could limit the ability to hypothesise replicability of results. Finally, while this study explored the feasibility of the *Work and Cancer* intervention online, it cannot be concluded that it would be feasible face to face.

## Conclusion

This study has shown it is feasible to recruit and retain participants to the 6-week intervention which was widely accepted by women living with and beyond breast cancer navigating return to work or work maintenance. This study builds on the limited research available in work-focused interventions for women living with and beyond breast cancer, and findings support the necessity for further testing of an intervention of this kind. Minor amendments such as increasing session length and offering outcome measures in other formats such as post and/or online survey will be made. A definitive intervention trial is currently underway to assess the effectiveness of the intervention for women with breast cancer and based on findings, future studies could assess transferability of the intervention for other cancer types.

**Key findings**

- It is feasible to recruit and retain women with breast cancer to a work-focused intervention.
- A large-scale evaluation determining effectiveness of the ‘Work and Cancer’.
- Intervention is warranted.

**What the study has added**

This study has demonstrated that the *Work and Cancer* intervention is both feasible and acceptable to women living with and beyond breast cancer, and a large-scale evaluation is warranted.

## Supplemental Material

sj-doc-1-bjo-10.1177_03080226251319900 – Supplemental material for Feasibility and acceptability of a self-management intervention supporting return to work for women with breast cancer Supplemental material, sj-doc-1-bjo-10.1177_03080226251319900 for Feasibility and acceptability of a self-management intervention supporting return to work for women with breast cancer by Naomi Algeo, Kathleen Bennett, Louise Brennan and Deirdre Connolly in British Journal of Occupational Therapy

sj-doc-2-bjo-10.1177_03080226251319900 – Supplemental material for Feasibility and acceptability of a self-management intervention supporting return to work for women with breast cancer Supplemental material, sj-doc-2-bjo-10.1177_03080226251319900 for Feasibility and acceptability of a self-management intervention supporting return to work for women with breast cancer by Naomi Algeo, Kathleen Bennett, Louise Brennan and Deirdre Connolly in British Journal of Occupational Therapy

sj-docx-3-bjo-10.1177_03080226251319900 – Supplemental material for Feasibility and acceptability of a self-management intervention supporting return to work for women with breast cancer Supplemental material, sj-docx-3-bjo-10.1177_03080226251319900 for Feasibility and acceptability of a self-management intervention supporting return to work for women with breast cancer by Naomi Algeo, Kathleen Bennett, Louise Brennan and Deirdre Connolly in British Journal of Occupational Therapy

sj-docx-4-bjo-10.1177_03080226251319900 – Supplemental material for Feasibility and acceptability of a self-management intervention supporting return to work for women with breast cancer Supplemental material, sj-docx-4-bjo-10.1177_03080226251319900 for Feasibility and acceptability of a self-management intervention supporting return to work for women with breast cancer by Naomi Algeo, Kathleen Bennett, Louise Brennan and Deirdre Connolly in British Journal of Occupational Therapy
