# Supplementary material for: Feasibility and acceptability of a self-management intervention supporting return to work for women with breast cancer
Source: Br J Occup Ther. 2025 Mar 20;88(7):429–43. doi: 10.1177/03080226251319900 (PMC12207618; doi:10.1177/03080226251319900)
Supplement: sj-docx-3-bjo-10.1177_03080226251319900 – Supplemental material for Feasibility and acceptability of a self-management intervention supporting return to work for women with breast cancer [file sj-docx-3-bjo-10.1177_03080226251319900.docx]

**INTERVIEW SCHEDULE**

| **General** |
| --- |
| What were your overall thoughts of the programme?  *Prompt:* What did you like/dislike? What would you change about the programme? What motivated you to participate and stay on in the programme? |

| **Acceptability of the Intervention** |
| --- |
| **Format:**   - This was an online programme. What are your thoughts on this type of format? Would you have preferred face-to-face? Why? Why not?   **Hosting:** This programme had a blended format in that it was both group-based with an individual component. What did you think about this format?  **Timing:**   - You have [not yet returned to work/returned to work]. When do you think would be the best time to offer this type of programme? - *If applicable:* There were some participants who had already returned to work. Did you find the mix useful or should the programme be more focused to those who have yet to return? - Sessions were 90 minutes online. Was this too long, too short or just right? Why? - The programme was 6 weeks in length. Was this too long, too short or just right? Why?   **Setting:**   - This programme was hosted online using Zoom. What do you think of this platform? Would you have preferred to use a different online platform? Why? Why not? - If this programme was to be hosted face-to-face, where would it be best set?   **Content:**   - Was there any content that you felt was missing? What was it? - Was there any content that you felt was irrelevant? What was it? - What content of the programme did you think was most important? - What did you think about the order in which the modules were presented? - What were your thoughts on the letter to the employer? - What were your thoughts on the return-to-work/work maintenance plan report?   **MDT Facilitators:** This programme was OT-led with multidisciplinary team input from a community welfare officer and physiotherapist. What did you think of this format?  **Theory:** What were your thoughts on the goal-setting aspect? Did you find having others in the group motivating or unmotivating for you? Why? Why not? |

| **Acceptability of Outcome Measures** |
| --- |
| **Show questionnaires up on screen to prompt participant*  Thinking about the EORTC QOL questionnaire, did you think this was: (i) easy or difficult to understand? (ii) covered most/all aspects of quality of life?  Thinking about the Employment questionnaire, did you think this was: (i) easy or difficult to understand? (ii) covered most/all aspects of your working status?  Any other comments about the questionnaires? |

| **Facilitators and Barriers to completing the Intervention** |
| --- |
| **Facilitators:** What helped you to be able to complete the programme?  **Barriers:** Was there anything that made it difficult for you to complete the programme? |

| **Reflection** |
| --- |
| - Has your approach to your return to work changed at all since starting the programme? If so, how? - Moving forward, what are your thoughts about this programme being potentially rolled out further in the future? - Any final thoughts about the programme, or anything that you wanted to mention but haven’t had the opportunity yet? |
